# A Novel Machine Learning Technique for Fault Detection of Pressure Sensor

**DOI:** 10.3390/e27020120

**Published:** 2025-01-24

**Authors:** Xiufang Zhou, Aidong Xu, Bingjun Yan, Mingxu Gang, Maowei Jiang, Ruiqi Li, Yue Sun, Zixuan Tang

**Affiliations:** 1 Key Laboratory of Networked Control Systems, Chinese Academy of Sciences, Shenyang 110169, China; zhouxiufang@sia.cn (X.Z.); yanbingjun@sia.cn (B.Y.); gangmingxu@sia.cn (M.G.); liruiqi1@sia.cn (R.L.); tangzixuan@sia.cn (Z.T.); 2 Shenyang Institute of Automation, Chinese Academy of Sciences, Shenyang 110169, China; 3 University of Chinese Academy of Sciences, Beijing 100049, China; 4 Institute of Future Human Habitats, Tsinghua Shenzhen International Graduate School, University Town of Shenzhen, Nanshan District, Shenzhen 518055, China; jiangmaowei@sia.cn; 5 School of Information Science and Engineering, Shenyang University of Technology, Shenyang 110870, China; sunyue@sut.edu.cn

**Keywords:** pressure transmitter, sensing line, fault detection, machine learning, XGBoost

## Abstract

Pressure transmitters are widely used in the process industry for pressure measurement. The sensing line, a core component of the pressure sensor in the pressure transmitter, significantly impacts the accuracy of the pressure transmitter’s output. The reliability of pressure transmitters is critical in the nuclear power industry. Blockage is recognized as a common failure in pressure sensing lines; therefore, a novel detection method based on Trend Features in Time–Frequency domain characteristics (TFTF) is proposed in this paper. The dataset of pressure transmitters comprises both fault and normal data. This method innovatively integrates multi-scale time series decomposition algorithms with time-domain and frequency-domain feature extraction techniques. Initially, this dataset is decomposed into multi-scale time series to mitigate periodic component interference in diagnosis. Subsequently, via the sliding window algorithm, both the time-domain features and frequency-domain features of the trend components are extracted, and finally, the XGBoost algorithm is used to detect faults. The experimental results demonstrate that the proposed TFTF algorithm achieves superior fault detection accuracy for diagnosing sensing line blockage faults compared with traditional machine learning classification algorithms.

## 1. Introduction

In industrial production, process transmitters supply field signals to the control system, enabling monitoring of process parameters throughout operational stages [1]. Pressure transmitters, which are among the most important field devices, are widely used and significantly impact the stability and safety of factory production. The nuclear power industry, in particular, explicitly requires reliable pressure transmitter operation [2].

**Figure 1:**
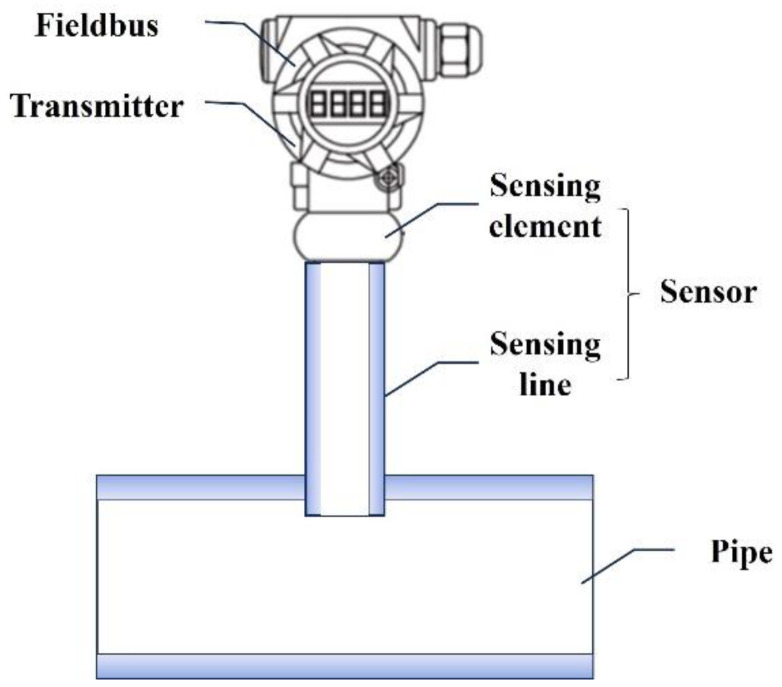
Diagram of pressure transmitter components.

As shown in Figure 1, pressure transmitters have three main components: a sensor, a transmitter, and a fieldbus [3]. Timely and accurate detection and diagnosis of potential faults are crucial for ensuring stable and safe production, given the importance of automated monitoring. While transmitters and fieldbuses already have mature fault detection techniques, effective digital fault detection methods for sensors are limited because of their working principle of converting physical signals into analogue electrical signals.

**Figure 2:**
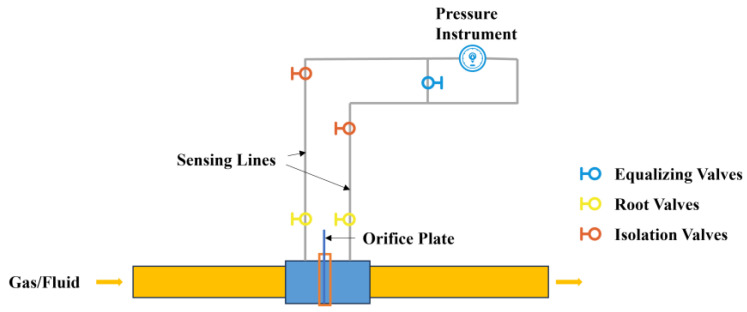
Structural diagram of the sensing line.

The pressure sensor comprises a sensing line and a pressure-sensitive element. To increase the operational stability and extend the service life of the pressure transmitter, the pressure-sensitive elements and transmitters are usually located away from the pipeline measurement point, whereas the sensing line is placed directly in the harsh environment [1], as shown in Figure 2. The sensing line serves as a critical link between the measurement point and the pressure-sensitive element. Abnormal conditions such as blockage, freezing, air bubbles, and leakage with the sensing line can severely impact the accuracy of the pressure signal measurement, potentially leading to delayed or distorted readings. To address sensing line blockage faults, this paper proposes a simple yet effective sensor fault detection method that directly utilizes pressure process data, aiming to ensure reliable pressure transmitter operation.

An analysis of the Licensee Event Report database by the U.S. Nuclear Regulatory Commission reveals that 30% of sensing line problems result from aging issues [4], with over 60% of these problems attributed to blockages [5]. For example, in nuclear power plants, the solidification and accumulation of boron, sludge, and other contaminants in pipes often block sensing lines [6]. The UCREG/CR-5851 report [7] highlights that sensing line blockage is a critical failure mode in nuclear power plant operations, resulting in pressure measurement errors [8] and posing a significant threat to industrial safety. To ensure accurate pressure transmitter signal output, routine practices such as periodic pipe flushing, backfilling, or draining are performed to remove deposits from the sensing line [7,9]. These regular maintenance activities necessitate operational interruptions, resulting in production downtime and significantly elevated time and economic costs. Thus, an urgent need exists for online detection of sensing line blockages.

Currently, diagnostic techniques for sensing line blockage are classified into two categories: mechanism-based techniques and data-driven techniques. In mechanism-based techniques, equivalent circuit models are commonly employed for sensing line fault detection. Kang Lin et al. [10,11] and Mangi et al. [12] analysed the impact of blockages on the transfer function of pressure systems through the construction of equivalent circuit models. However, the effectiveness of this method is fully dependent on the accuracy of the equivalent circuit model, which requires substantial subjective input from experts, thereby introducing potential errors. Various data-driven methods have been proposed. Hashemian [5] introduced an analytical method to evaluate the dynamic characteristics of pressure sensors, specifically the ‘response time’. In this method, noise signals are extracted from pressure data and subsequently utilized to derive response time features, which are then analysed to evaluate the fault status of the sensing lines on the basis of changes in response time, thereby achieving fault detection. He et al. [13] developed a method that leverages one-dimensional convolutional neural networks. Feature extraction from noise signals within pressure transmitter data was conducted via convolutional layers, followed by classification of different blockage states through fully connected and output layers. However, both of these data-driven methods are susceptible to strong external noise interference, which affects the accuracy of the diagnosis. To improve the diagnostic accuracy of sensing line blockages, Jianqiu Zhang et al. [14] applied the wavelet transform algorithm to perform time-frequency analysis on process pressure data, thereby reducing the impact of external noise on the diagnostic results. However, selecting an appropriate wavelet basis function and determining the number of decomposition levels in practice still present challenges [14]. Tabaru et al. [15] employed a mathematical statistics-based approach to detect blockages by analysing pressure fluctuation frequencies, noting that these frequencies decreased upon blockage occurrence. However, this method is highly sensitive to changes in the working environment, requiring frequent calibration of the diagnostic threshold. Despite advancements in diagnostic techniques for sensing line blockage, accurately detecting these blockages remains challenging. These limitations in existing methods highlight the need for a more effective and robust approach to accurately detect sensing line blockages. The limited availability of publicly accessible experimental datasets hinders the in-depth exploration of diagnostic techniques in this field.

To address these challenges, this paper adopts a data-driven approach. Unlike existing methods that rely mainly on noise signals or are highly sensitive to environmental changes, this paper proposes a novel sensing line fault detection method based on Trend Features in the Time–Frequency domain (TFTF). This method employs a multi-scale time series decomposition algorithm to separate the signal into trend and periodic components. Time- and frequency-domain features are then extracted from the trend components, effectively improving the accuracy and robustness of the diagnostic method, with the XGBoost algorithm applied for classification to achieve fault detection. The main contributions of this paper are summarized as follows:

1. To enhance feature extraction from pressure transmitter data, a fault detection method based on Trend Features in Time–Frequency domain (TFTF) is proposed. This method utilizes a multi-scale time series decomposition algorithm to separate process data into periodic and trend components.
2. To comprehensively capture trend component information, feature extraction methods in both the time- and frequency-domains are introduced, significantly enhancing fault detection accuracy.
3. A sensor failure simulation platform was constructed to collect critical data, and a specialized test dataset was created. Various algorithms were applied to analyse this dataset, and the results demonstrated that the TFTF algorithm achieves high fault detection accuracy, reaching 99.39%.

The structure of this paper is organized as follows. The proposed sensing line fault detection method is detailed in Section 2. Section 2.1 presents a multi-scale time series decomposition algorithm that decomposes the collected sensor signals into distinct trend and periodic components. The process for extracting time-domain and frequency-domain features is thoroughly introduced in Section 2.2. The principles underlying the XGBoost algorithm are discussed in Section 2.3. Section 3 introduces the experimental platform and the constructed dataset. Section 4 describes the experimental results. In Section 4.1, the proposed method is compared with commonly used classification techniques. Section 4.2 presents three sets of comparative experiments conducted across different dimensions. Section 4.3 covers the feature set screening process. Section 5 presents the discussion. Section 6 presents the paper’s conclusions and provides an outlook on future research directions.

## 2. Method

**Figure 3:**
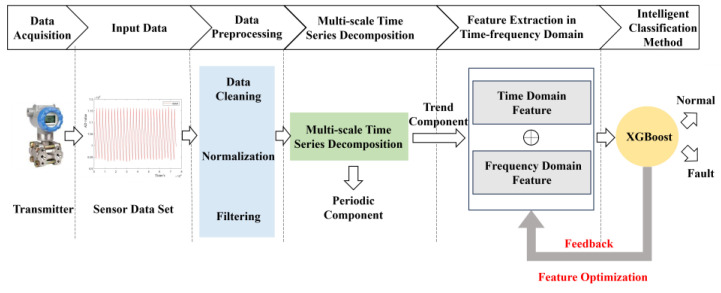
The architecture of the fault detection method based on Trend Features in Time–Frequency domain.

A novel diagnostic method utilizing Trend Features in the Time–Frequency domain (TFTF) is proposed to improve sensor fault detection accuracy. The processing steps of the method are illustrated in Figure 3. Through fault injection, both normal and faulty pressure sensor signals are collected in the laboratory. The data are preprocessed, including data cleaning, normalization, and filtering. A multi-scale time series decomposition algorithm is employed to separate the signal data into trend and periodic components. Time- and frequency-domain features are extracted from the trend components, followed by classification of the feature set via the XGBoost algorithm to diagnose abnormal conditions. XGBoost is a machine learning algorithm capable of capturing nonlinear relationships within data and providing measures of feature importance, demonstrating robust performance across diverse datasets. The TFTF method provides a novel solution for industrial fault detection.

### 2.1. Multi-Scale Time Series Decomposition

Sensing line blockage can alter the dynamic characteristics of the response time in pressure sensors, leading to long-term trends that cause errors in the pressure output signal [7]. Repetitive patterns of periodic signals can obscure trend information that indicates changes in system health, thereby interfering with the identification of gradual faults. This paper employs a multi-scale time series decomposition algorithm to decompose the pressure sensor’s output signal, removing periodic components while retaining trend components. Subtle changes within the trend components serve as a basis for fault detection. An in-depth analysis and detailed explanation of the trend components are provided in Section 4.2.1.

In earlier time series decomposition techniques, Wu and Zeng utilized moving averages to reduce periodic oscillations and emphasize long-term trends [16,17]. The algorithm for time series $X\in R^{I\times q}$ is as follows:

(1) $$X_{p(i)}=Padding{\left(X\right)}_{kernel\_p\left(i\right)}$$

(2) $$X_{trend\left(i\right)}={Avgpool(X_{p(i)})}_{kernel\_a(i)}$$

(3) $$X_{periodic\left(i\right)}=X-X_{trend\left(i\right)}$$

(4) $$kernel\_a(i)=kernel\_p\left(i\right)-1$$

Here, $R^{I\times q}$ represents the set of real numbers (R) arranged in I rows and q columns. $X_{p(i)}$ represents the time series padded (Padding(·)) using different sizes of kernels ($kernel\_p\left(i\right)$). The trend component is represented by $X_{trend(i)}\in R^{I\times q}$, and the periodic component is represented by $X_{periodic(i)}\in R^{I\times q}$. Padding(·) and Avgpool(·) keep the length of the time series unchanged. The purpose of (Avgpool(·)) is to reduce data dimensionality by calculating the average within a fixed-size window (kernel_a(i)), referred to as average pooling. However, $kernel\_p\left(i\right)$ is determined by kernel_a(i) and is artificially predetermined, which can lead to significant variations between the trend and periodic components derived from different kernels. The selection of the size of the pooling kernels is based on experiments and experience, and the specific experimental results can be found in Appendix C.

**Figure 4:**
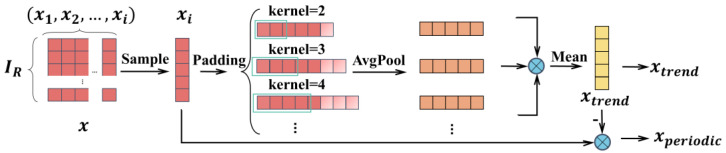
Multi-scale time series decomposition algorithm.

Moreover, complex periodic and trend components are frequently observed in real-world production environments. Accurately extracting these components via a single fixed-window average pooling algorithm presents a significant challenge. To address this issue, this paper proposes a multi-scale time series decomposition algorithm that incorporates a series of average pooling kernels of varying sizes. By selecting different average pooling kernels, multiple trend components are extracted from the raw data. Section 4.2.2 provides a detailed analysis of the advantages of using the multi-scale time series decomposition algorithm for fault detection. The results indicate a significant improvement in fault detection accuracy following the application of the multi-scale time series decomposition algorithm (Figure 4). This algorithm combines the extracted trend components by scaling factors derived from preliminary experiments to form the final trend component. Specifically, for the raw input $X\in R^{I\times q}$, the processing steps are as follows:

(5) $$X_{trend}=mean\left(V\left(X\right)\cdot X_{trend\left(i\right)}\right)$$

(6) $$X_{periodic}=X-X_{trend}$$

Here, X is the input time series, $X\in R^{I\times q}$. $V\left(X\right)$ denotes the scaling factors used to combine the different trend components.

### 2.2. Feature Extraction in the Time-Frequency Domain

**Figure 5:**
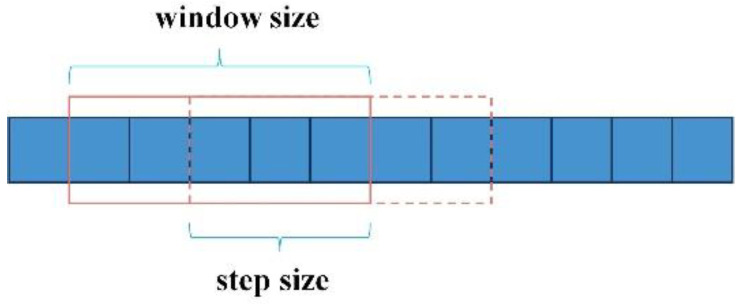
Sliding window algorithm.

To analyse the characteristics of the trend components in the sensor signals, the sliding window algorithm (as shown in Figure 5) is employed to divide the trend components into appropriately sized segments [18]. Time-domain and frequency-domain features are extracted from each data segment [19,20,21] to create a feature dataset, which provides an information source for the fault detection of the sensing line.

Through multiple experiments, it was concluded that setting the window size to 2000 and the stride to 1000 achieves a balance between accuracy and feature representation capability. This configuration effectively captures trend information while avoiding excessive redundancy.

#### 2.2.1. Extraction of Time-Domain Features

Time-domain features provide the statistical characteristics and morphological attributes of signals in the time domain. A series of mathematical transformations are applied to extract 10 time-domain features of the signal: peak-to-peak value, amplitude, variance, root mean square, kurtosis, average absolute value, waveform factor, peak factor, impulse factor, and margin factor, as shown in Table 1. Through comprehensive analysis, the fundamental properties of the signal, such as strength, waveform, and periodicity, can be thoroughly described. In Table 1, $X_{i}\in X_{trend}\;$represents the i-th signal’s sample points, N denotes the total number of samples, $\bar{X}$ indicates the mean value of the signal, and s represents the standard deviation of the samples.

#### 2.2.2. Extraction of Frequency-Domain Features

The frequency-domain features illustrate the distribution and characteristics of a signal within the frequency domain. These characteristics provide essential spectral information for classification purposes. Through the application of the Fast Fourier Transform, the time-domain signal is converted into a frequency-domain signal, allowing for the extraction of frequency-domain information.

The Fast Fourier Transform (FFT) exploits the periodicity and symmetry of the weighting function $W_{N}^{kn}$ to perform a series of decomposition and recombination on a signal of length *N*. As a result, the number of computations involved in the Discrete Fourier Transform (DFT) is reduced, thereby increasing the calculation speed.

(7) $$X\left(k\right)=\sum_{n=0}^{N-1}x\left(n\right)W_{N}^{kn}\left(k=0,1,\ldots ,N-1,W_{N}=e^{-j\frac{2\pi }{N}}\right)$$

In this context, $X\left(k\right)$ represents the data following the FFT transformation; x(n) denotes the signal to be transformed; *N* indicates the number of sampling points of the signal; and $W_{N}^{kn}$ corresponds to the weight function of the Discrete Fourier Transform (DFT).

On the basis of the data derived from the Fourier transformation, eight frequency-domain features are further extracted, as shown in Table 2. These include the frequency-domain amplitude mean value, gravity frequency, mean square frequency, root mean square frequency, frequency variance, average frequency, total power, and average power. In Table 2, X(k) refers to the spectral component of the signal after performing the Fast Fourier Transform (FFT). Specifically, $f_{k}$ represents the sampling frequency, and X(k) denotes the frequency component corresponding to index k in the frequency spectrum. N is the total number of spectral components.

Compared with techniques that omit extracted features and rely solely on single-domain features, methods that combine both time-domain and frequency-domain features capture the intrinsic characteristics of the signal more comprehensively. This approach enhances the feature representation of the signal, leading to improved classification results. Refer to Section 4.2 for a detailed analysis and corresponding experimental results.

### 2.3. Intelligent Classification Method

After the characteristics of the signal’s trend components are extracted, classification is required for normal diagnosis. This task falls under the category of supervised learning classification. Common classification algorithms include logistic regression, K-nearest neighbor algorithm, support vector machines, XGBoost, Random Forest, neural network, etc. In this paper, the XGBoost (eXtreme Gradient Boosting) algorithm is employed for fault detection. This model is a machine learning algorithm based on gradient boosting and is designed to address regression and classification problems in supervised learning. The XGBoost algorithm enhances model accuracy by iteratively adding new decision trees that correct errors from previous rounds. Gradient boosting serves as the foundation of the XGBoost algorithm. This approach considers both prediction error and model complexity, helping to prevent overfitting while enhancing prediction accuracy. The main process of the XGBoost algorithm is as follows.

The objective function of the XGBoost algorithm consists of two components: the loss function and the regularization term, as represented in the following formula.

(8) $$L\left(\Phi \right)=L\left(\Phi \right)+\Omega \left(\Phi \right)$$

where Φ represents the parameters obtained from training on the given data. L denotes the loss function, which measures the model’s degree of fit. Ω denotes the regularization term, which is used to assess the model’s complexity. Assuming that the model consists of k decision trees, with input $X=\{F_{1},{\;F}_{2},\ldots F_{17},{\;F}_{18},{\;F}_{19}\}$, the output ${\hat{y}}_{i}=\{0,1\}$ is derived from the ensemble of decision trees through voting or averaging.

(9) $${\hat{y}}_{i}=\sum_{j=1}^{k}f_{j}\left(x_{i}\right),f_{j}\in F$$

Here, $x_{i}$ represents the i-th sample, with $x_{i}\in X$; $f_{j}\left(x_{i}\right)$ represents the model of the *j*-th tree; and F represents the collection of k decision tree models.

The objective function can be expressed as:

(10) $$L\left(\Phi \right)=\sum_{i=1}^{n}l\left(y_{i},{\hat{y}}_{i}\right)+\sum_{j=1}^{k}\Omega \left(f_{j}\right)$$

Here, *n* represents the number of samples, and $y_{i}$ represents the true value of the sample.

The weight of leaf node j: $w_{j}^{*}=-\frac{G_{j}}{H_{j}+\lambda }$

The optimal solution of the objective function:$\;L^{{\left(k\right)}^{*}}=-\frac{1}{2}\sum_{j=1}^{T}\frac{{G_{j}}^{2}}{H_{j}+\lambda }+\gamma T$

The sum of the first-order partial derivatives of the samples contained in leaf node *j*, denoted as $G_{j}$, is a constant: $G_{j}=\sum_{i\epsilon I_{j}}g_{i}$. The sum of the second-order partial derivatives of the samples contained in leaf node j, denoted as $H_{j}$, is a constant: $H_{j}=\sum_{i\epsilon I_{j}}h_{i}$. *T* denotes the number of leaf nodes, and γ and λ represent the regulation functions of the corresponding terms.

The optimization of the objective function is reformulated to find the minimum value of a quadratic function. Following a node split in the decision tree, the change in model performance is evaluated on the basis of the objective function. If model performance improves post-split, the modification is adopted; otherwise, the split is terminated. Additionally, the regularization applied in optimizing the objective function helps prevent overfitting during training.

In this study, the machine learning model was trained on Python 3.11.4 via scientific computing libraries such as Numpy 1.25.0 and Pandas 2.1.4, which provide efficient data structures and preprocessing techniques. Scikitlearn 1.2.2 and Xgboost 2.0.3 were used to implement the SVM, RF, and XGBoost algorithms [22].

We utilized Optuna, a hyperparameter optimization framework, to search efficiently for the optimal combination of hyperparameters. Optuna’s tree-structured Parzen estimator (TPE) algorithm uses distribution modelling and objective function evaluation, enabling it to find superior solutions in high-dimensional hyperparameter spaces with a greater efficiency than grid search or random search.

The dataset was divided into a training set and a validation set, with the log-loss of the validation set used as the objective function. During the tuning process, Optuna dynamically adjusts the hyperparameter combinations by training the model on the training set and evaluating the loss on the validation set. We conducted 50 experiments, with each experiment testing a new hyperparameter combination. To accelerate convergence, an early stopping strategy was employed, halting training if the validation performance did not improve significantly over 10 consecutive iterations.

While the tuning process identified the hyperparameter combination that performed best on the validation set, we observed that these parameters led to an excessively large model size, increasing the risk of overfitting. To address this, we opted for the second-best set of hyperparameters, achieving a balance between performance and model complexity. This choice ensured a trade-off that enhanced the model’s generalization ability on the test set.

To mitigate overfitting risk further, we divided the training data into training and validation sets and monitored the log-loss of the validation set during training. If the validation log-loss did not improve significantly over 10 consecutive iterations, training was terminated. This early stopping mechanism effectively prevented the model from overfitting to the training data and ensured robust performance on the validation set.

The following parameter settings were selected to optimize model performance: the tree depth (*max_depth*) was set to 3 to prevent overfitting, and the learning rate (*learning_rate*) was set to 0.1 to control the step size of each iteration. The size of the weak classifiers (*n_estimators*) was set to 100. The objective function was set to ‘*binary: logistic*’ for binary classification problems. The sample proportion used for training each tree (*subsample*) was set to 0.8, the proportion of features randomly selected for constructing each tree (*colsample_bytree*) was set to 0.8, and the minimum sum of sample weights required in a leaf node (*min_child_weight*) was set to 1. Furthermore, the regularization parameters *reg_alpha* and *reg_lambda* were set to 0 and 1, respectively, to control model complexity. The experimental results indicate that these parameters enable the model to achieve satisfactory performance on the given dataset.

## 3. Experiment Platform

**Figure 6:**
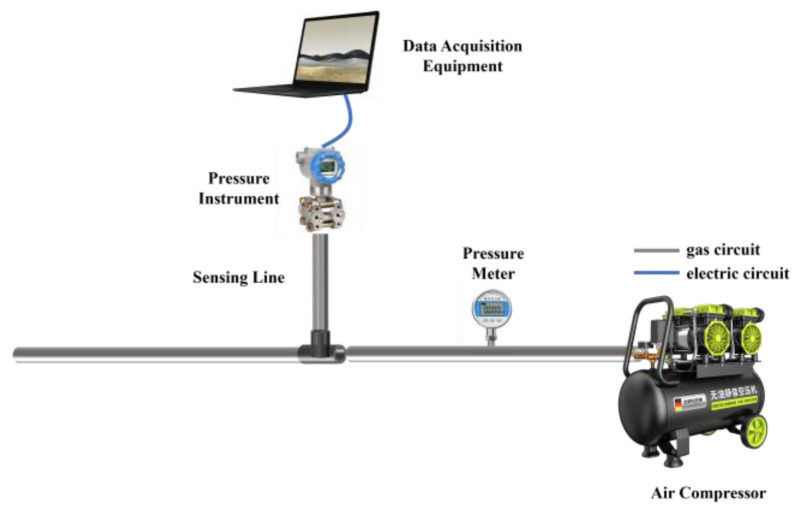
The experimental platform simulates sensing line problems.

**Figure 7:**
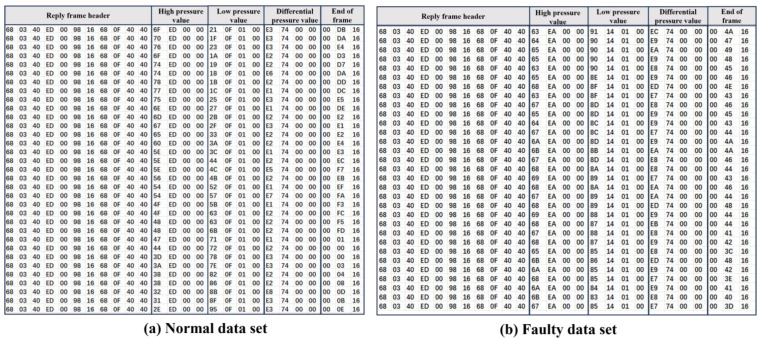
Datasets of the pressure sensor.

In this study, a pressure sensor fault simulation platform consisting of an air compressor, a pressure transmitter, a data acquisition device, and other components was established. The pressure transmitter is connected to the main pipeline system through a T-joint, facilitating precise monitoring of pressure changes within the pipeline system, as shown in Figure 6. In this study, the data sampling rate was set at 20 Hz, meaning that 20 pressure signal points were collected per second. Blockage faults within the pressure sensing line are simulated via fault injection. To simulate sensing line blockage faults, we controlled the occurrence and severity of faults on the experimental platform by varying the diameter of the sensing line. The injection process was highly controllable, ensuring the reliability and consistency of the experimental results. Fault states were identified on the basis of changes in pressure signal characteristics. During the experiments, the gel was incrementally injected, and real-time monitoring of pressure variations was conducted to observe significant differences between the normal and blockage states. After each gel injection, the corresponding state (normal or fault) was labelled on the basis of experimental records and real-time pressure signal changes. To ensure labelling accuracy, the collected signals were further manually verified after the experiments. Under normal conditions, there is no gel injection, the pressure lead tube is unblocked, and the pressure signal is stable. A fault state is an abnormal pressure signal after gel injection, such as a longer response time. To simulate realistic pressure conditions, an air compressor was used to apply pressure, accurately reproducing the dynamic pressure environment commonly found in industrial systems. The platform can simulate various working states of the pressure sensing line, encompassing both normal and specific fault conditions. Blockage faults within the pressure sensing line are simulated via fault injection. The key components of the platform and their parameters are provided in Table 3. On the basis of this setup, a dataset containing 45 groups of experimental data was constructed, with each group comprising approximately 75,000 sampling points, resulting in a total of over 3,375,000 records. This dataset includes sensor data representing both the normal and faulty states of the pressure sensing line. Table 4 shows that the normal dataset contains 1,725,000 rows, whereas the faulty dataset includes 1,650,000 rows. Sample data from these sets are depicted in Figure 7.

## 4. Results

### 4.1. Main Experimental Results

**Figure 8:**
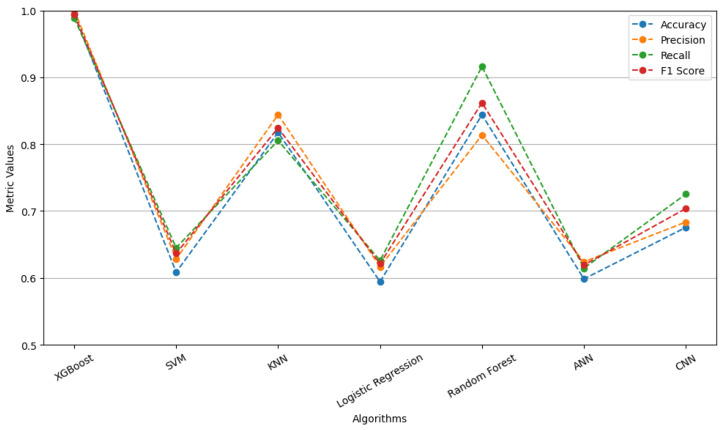
Performance metrics comparison across algorithms.

A comparative analysis was conducted to evaluate the performance of various machine learning algorithms in diagnosing blockage faults within the sensing lines of pressure transmitters. The selected algorithms included SVM, k-Nearest Neighbors, Logistic Regression, Random Forest, ANN, and CNN [23,24,25,26]. The dataset collected from the experimental platform (Figure 3) was divided into training and testing sets at an 8:2 ratio. Each model’s performance was assessed and compared via metrics such as accuracy, recall, F1 score, and precision. The performance of these models on the test set is displayed in Figure 8. The results indicate that the XGBoost algorithm achieves the highest scores across all the evaluation metrics, notably attaining a test accuracy of 99.39%. The test accuracies of all the algorithms are presented in Table 5. To verify the robustness of our model on small-scale datasets and its applicability in scenarios with limited data, we conducted a series of comparative experiments. The results of these experiments are detailed in Appendix A. Furthermore, in Appendix B, we discuss the sensitivity of the proposed method to noise.

**Figure 9:**
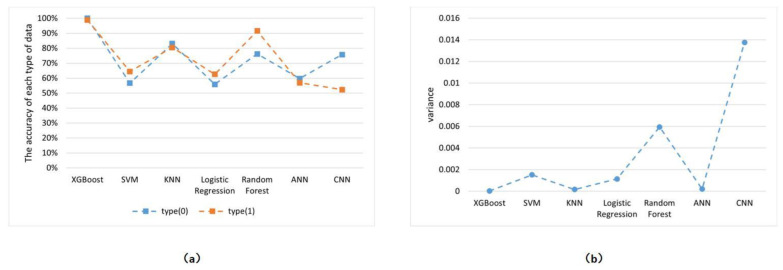
(**a**) Test accuracy of each algorithm on two types of data. (**b**) The variance in the accuracy of each algorithm on two types of data.

In this study, the aforementioned algorithms were tested to assess the robustness of various models in handling the sensing line dataset. The test accuracy of these models in distinguishing between the normal and faulty states of the sensing line is presented in Figure 9a. In this figure, 0 and 1 represent the two working states of the sensing line, corresponding to normal and faulty conditions, respectively. Furthermore, the variance in test accuracy for each category of data across the seven algorithms is depicted in Figure 9b.

As shown in Figure 9a, the test accuracy of the XGBoost algorithm exceeds 99% for datasets in both normal and faulty states. This exceptionally high accuracy indicates the reliability of the XGBoost algorithm in handling such problems. Furthermore, as observed in Figure 9b, the variance in test accuracy for this algorithm across both dataset types is only 0.0000025, confirming the model’s robustness.

**Figure 10:**
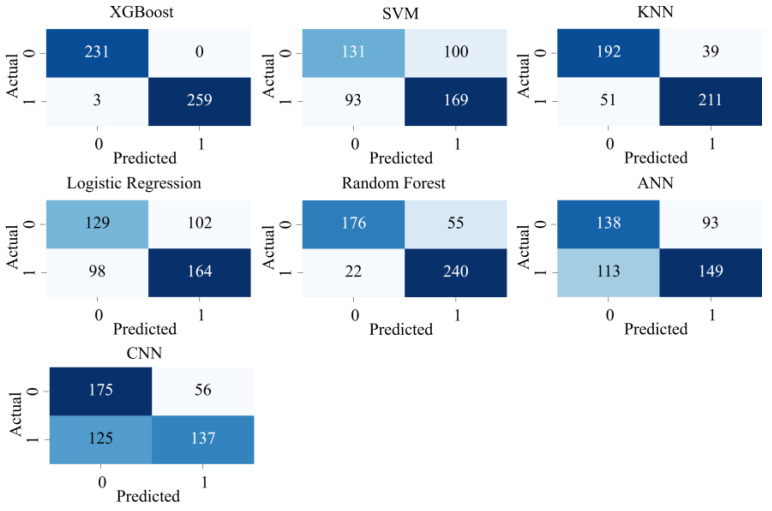
Confusion matrices of the seven algorithms.

The prediction results of XGBoost and six comparative algorithms on the test set are presented as confusion matrices in Figure 10. In these matrices, the *x*-axis represents the predicted labels, whereas the *y*-axis represents the true labels. The value at position (i, j) in the matrix indicates the number of samples with a true label of *j* that were predicted as *i*. The confusion matrix not only identifies the sample distribution across different categories in the test set but also provides a visual evaluation of the model’s predictive capabilities.

**Figure 11:**
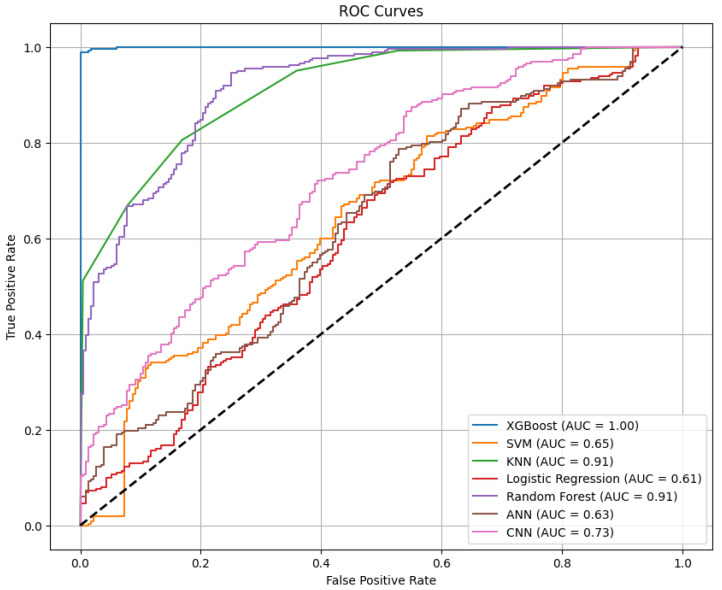
ROC curves of the seven algorithms.

**Figure 12:**
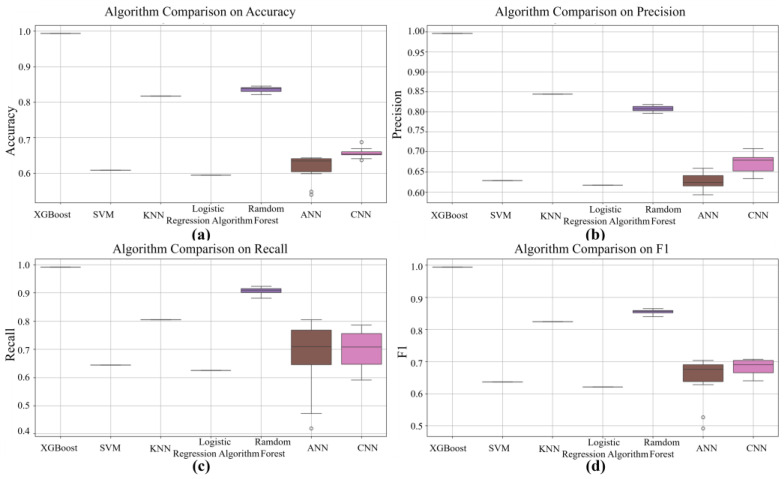
Boxplots of the performance differences of the seven algorithms.

Figure 10 shows that the XGBoost model achieves a prediction accuracy of 99%, further confirming its outstanding performance. In addition, the ROC (Receiver Operating Characteristic) curves, which are widely used for evaluating classification models, are displayed in Figure 11. Among the ROC curves of the seven algorithms, XGBoost has an AUC (Area Under the Curve) approaching 1, significantly outperforming the other six algorithms. In Figure 12, in the boxplots of the seven algorithms, the performance of XGBoost is clearly due to the other six algorithms. This demonstrates the superior classification ability and robustness of XGBoost, making it particularly effective in the context of this study.

By comparing the performance of multiple algorithms across four key metrics—accuracy, precision, recall, and F1 score—the superiority of our proposed method is clearly demonstrated. The results reveal that the mean performance of our method across all the metrics is close to 1.0, significantly outperforming the other algorithms. Furthermore, the boxplot results show that the performance variation of our model is nearly negligible, highlighting its superior stability compared with other algorithms. This demonstrates that our method not only achieves a leading position in overall performance but also maintains exceptional robustness under varying conditions, such as changes in random seeds and data splits.

In contrast, Random Forest, as the second-best traditional machine learning algorithm, performs relatively well across all four metrics. However, its mean precision, recall, and F1 score remain slightly lower than those of our model, with marginally greater performance variation. Additionally, deep learning models, such as CNN and ANN, exhibit some instability, particularly ANN, which show significant fluctuations in recall and F1 scores, with some runs yielding low results. This instability reduces their reliability in practical applications. Traditional algorithms, such as SVM and Logistic Regression, perform considerably worse than our method does, with mean accuracy and precision scores below 0.65, indicating their limited suitability for the current task.

The *p* values for all four metrics are far below 0.05, confirming that the performance distributions of the different algorithms are significantly different. These differences are not due to random errors but are meaningful and statistically significant.

Traditional methods (SVM, KNN, Logistic Regression, Random Forest) rely on statistical features directly extracted from raw data, which fail to adequately capture trend information and time-frequency characteristics. In contrast, the TFTF method extracts both time-domain and frequency-domain features from trend components through multi-scale decomposition, significantly improving classification performance. While SVM and Logistic Regression perform well on linear data, they struggle with the nonlinear and complex distributions commonly found in industrial datasets. XGBoost, with its splitting strategy and weighted voting mechanism, handles nonlinear relationships more effectively, resulting in higher accuracy. ANN and CNN typically require a large amount of training data, which is often limited in industrial scenarios. XGBoost has a lower dependency on the data volume and efficiently utilizes the extracted features. While CNN automatically extract features, these features rely primarily on spatial structures and are less effective in capturing trend information in the time-frequency domain. The TFTF method, which involves pre-extracting specific features and leveraging XGBoost for classification, has clear advantages.

The decision tree structure in XGBoost naturally captures nonlinear relationships and trends within the data. By selecting optimal split points and leaf nodes, the decision tree can identify and learn the trend patterns within the data, allowing it to incorporate trend information into predictions. The regularization term in XGBoost effectively controls model complexity and mitigates overfitting. When handling trend data, proper regularization helps the model avoid overfitting noise while fitting the trend, thereby improving prediction accuracy.

### 4.2. Contrast Experiment

#### 4.2.1. Visualization Comparison of Trend and Periodic Components

**Figure 13:**
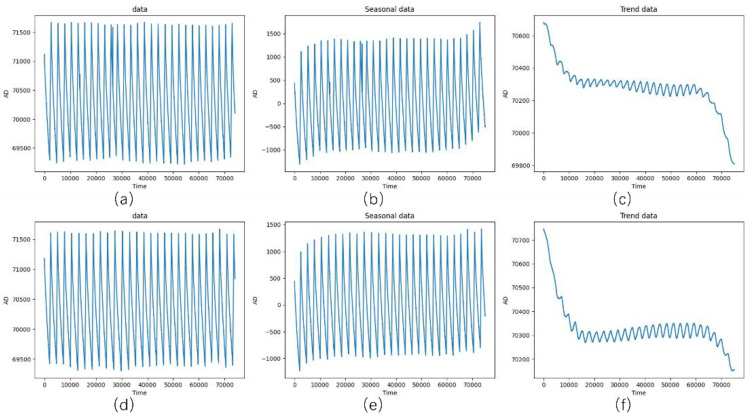
Visualization of periodic and trend components: (**a**) Data in the normal state, (**b**) Periodic data in the normal state, (**c**) Trend data in the normal state, (**d**) Data in the faulty state, (**e**) Periodic data in the faulty state, (**f**) Trend data in the faulty state.

During data visualization, the normal and faulty data of the sensing line exhibited a high degree of similarity. As shown in Figure 13a,d, identifying and distinguishing the working states directly from the raw data is challenging. By applying a time series decomposition algorithm to separate the original signal into periodic and trend components, the intrinsic features of the data are revealed.

In the visualization of the periodic component, signals in both the normal and faulty states maintain a certain degree of similarity, as shown in Figure 13b,e. This suggests that the periodic component may not contain key information for fault detection. Conversely, in the visualization of the trend component, as shown in Figure 13c,f, the signals in the normal and faulty states exhibit significantly different trends. This finding confirms that key information for fault detection resides in the trend component, reflecting long-term changes in the working state of the pressure transmitter.

#### 4.2.2. Comparison Between Single-Scale and Multi-Scale Time Series Decomposition Algorithms

A performance comparison between univariate and multivariate time series decomposition algorithms revealed that the multivariate decomposition algorithm separates the trend and periodic components of the time series more accurately. In practical applications, this method significantly improves fault detection accuracy compared with traditional univariate decomposition algorithms, as shown in Table 6. This improvement is attributed mainly to the ability of the multi-scale time series decomposition algorithm to identify and distinguish signal changes effectively across different time scales in complex time series data. This approach provides richer and more accurate information for fault detection.

#### 4.2.3. Analysis of Multiple Feature Processing Results

The results indicate that time-domain and frequency-domain features significantly enhance fault detection accuracy. When these two types of features are combined, particularly when the XGBoost algorithm is used as the classifier, the diagnostic accuracy increases by approximately 20%, as shown in Table 6. Furthermore, applying other classifiers also improves accuracy. This result confirms the effectiveness of combining time-domain and frequency-domain features.

### 4.3. Feature Selection

**Figure 14:**
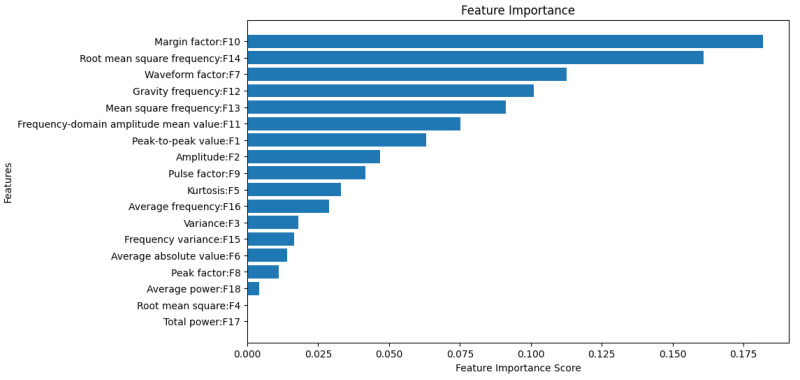
Feature importance map.

**Figure 15:**
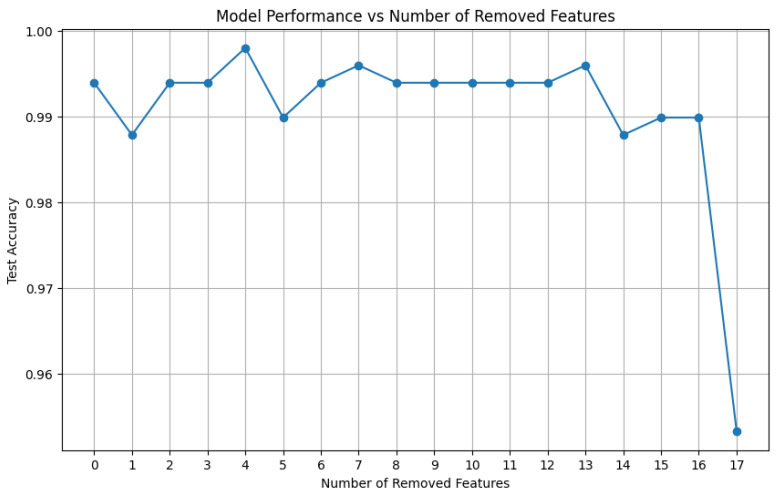
Model performance after removing features one by one.

During the TFTF training process, the XGBoost algorithm automatically evaluates the importance of the input features and generates corresponding feature importance scores. To accurately evaluate the importance of each feature in models that describe nonlinear relationships while ensuring the effective identification of features with the strongest distinguishing ability, Gain was selected as the feature importance evaluation metric in this study. As shown in Figure 14, the importance scores for the root mean square and total power are both 0. By successively deleting features in ascending order of their importance scores, the correspondence between the number of removed features and the removed features is shown in Table 7, and the change in model accuracy is shown in Figure 15. Notably, removing the four features with the lowest importance scores—total power, root mean square, average power, and peak factor—allows the model to achieve its highest accuracy of 99.79%.

This phenomenon can be attributed to redundant features diluting the model’s learning effectiveness and the weak correlation of these features with the target variable. These findings underscore the importance of iterative testing and feature optimization in achieving optimal model performance.

## 5. Discussion

Although this study focuses on the fault detection of pressure sensor sensing lines in nuclear power plants, the core techniques of the proposed method— time series decomposition, sliding window-based time, and frequency-domain feature extraction, and the XGBoost algorithm—are not dependent on the specific conditions of the nuclear power industry. Therefore, this method exhibits strong generalizability and can be applied to the fault diagnosis of pressure sensor sensing lines in other industrial settings.

In future work, we plan to expand the scope of fault diagnosis by considering a wider range of fault categories and moving beyond the traditional classification framework. Instead, we aim to explore fault diagnosis as a hypothesis testing problem. This shift could offer greater flexibility and generalizability, significantly improving the method’s performance in diverse and complex environments.

## 6. Conclusions

A novel detection method based on Trend Features in the Time–Frequency Domain (TFTF) is proposed in this paper to increase fault detection accuracy specifically for pressure sensor sensing line blockages. This method is tailored to address the challenges associated with identifying blockages in these lines. It employs a multi-scale time series decomposition algorithm to eliminate interference from periodic data within the operational environment. The time- and frequency-domain features of the trend components in the fault-injected data are extracted via a sliding window approach. Various machine learning techniques have been applied to classify time- and frequency-domain feature sets. The results indicate that the XGBoost algorithm has a significant advantage in terms of accuracy. Additionally, this method exhibits strong robustness. This method provides a set of approaches to ensure the high precision and reliability of pressure transmitters within the nuclear power industry. Future work will explore the applicability of this method under complex operational conditions and the potential for diagnosing additional faults in pressure sensors. The code is available at https://github.com/zxfsia96/pressure-sensor-fault-detection.

## Figures and Tables

**Table 1:** Time-domain features table.

Feature Name	Abbreviation	Formula	Meaning
Peak-to-peak value	$F_{1}$	$\max(X_{trend}$$)\text{–}\min(X_{trend}$)	The range of the signal value
Amplitude	$F_{2}$	$\max(X_{trend}$)	The strength of the signal
Variance	$F_{3}$	$\sqrt{\frac{1}{N}\sum_{i=1}^{N}{\left[X_{i}-\bar{X}\right]}^{2}}$	The degree of dispersion between signal data
Root mean square	$F_{4}$	$\sqrt{\frac{1}{N}\sum_{i=1}^{N}X_{i}^{2}}$	The degree of dispersion between signal data
Kurtosis	$F_{5}$	$\frac{N\left(N+1\right)}{\left(N-1\right)\left(N-2\right)\left(N-3\right)}\sum_{i=1}^{N}{\left(\frac{X_{i}-\bar{X}}{s}\right)}^{4}-\frac{3{\left(N-1\right)}^{2}}{\left(N-2\right)\left(N-3\right)}$	The smoothness of the signal waveform
Average absolute value	$F_{6}$	$\frac{1}{N}\sum_{i=1}^{N}\left|X_{i}\right|$	Amplitude of signal
Waveform factor	$F_{7}$	$\frac{F_{4}}{F_{6}}$	The smoothness and periodicity of the signal waveform
Peak factor	$F_{8}$	$\frac{F_{1}}{F_{4}}$	The extremity of the peak in the waveform
Pulse factor	$F_{9}$	$\frac{F_{1}}{F_{6}}$	The sharpness or impulsive characteristic of the signal
Margin factor	$F_{10}$	$\frac{F_{1}}{{\left(\frac{1}{N}\sum_{i=1}^{N}\sqrt{X_{i}}\right)}^{2}}$	The gap between the signal’s peak value and its mean level

**Table 2:** Frequency-domain features table.

Feature Name	Abbreviation	Formula	Meaning
Frequency-domain amplitude mean value	$F_{11}$	$\frac{1}{N}\sum_{k=1}^{N}X(k)$	The strength of frequency-domain signals
Gravity frequency	$F_{12}$	$\frac{\sum_{k=1}^{N}f_{k}X(k)}{\sum_{k=1}^{N}X(k)}$	Distribution of signal power spectrum
Mean square frequency	$F_{13}$	$\frac{\sum_{k=1}^{N}{f_{k}}^{2}X(k)}{\sum_{k=1}^{N}X(k)}$	Distribution of Main Frequency Bands in Signal Power Spectrum
Root mean square frequency	$F_{14}$	$\sqrt{F_{13}}$	Distribution of Main Frequency Bands in Signal Power Spectrum
Frequency variance	$F_{15}$	$\frac{\sum_{k=1}^{N}{{(f}_{k}-X_{12})}^{2}X(k)}{\sum_{k=1}^{N}X(k)}$	The degree of dispersion of the frequency distribution of the signal
Average frequency	$F_{16}$	$\frac{\sum_{k=1}^{N}f_{k}{X(k)}^{2}}{\sum_{k=1}^{N}{X(k)}^{2}}$	The center frequency or main frequency range of the signal
Total power	$F_{17}$	$\sum_{k=1}^{N}{X(k)}^{2}$	The total energy of the signal
Average power	$F_{18}$	$\frac{1}{N}\sum_{k=1}^{N}{X(k)}^{2}$	The average energy of the signal power spectrum

**Table 3:** Parameters of the core components of the experimental platform.

Device Name	Parameter Name	Parameter Value
Air compressor	Volume flow	100 L/min
	Maximum pressure	0.7 MPa
Pressure transmitter	Range	20.68 kpa–2068 kPa

**Table 4:** Dataset composition.

Types of Dataset	Data Size
Normal	1,725,000
Faulty	1,650,000

**Table 5:** Test set accuracy of the seven algorithms.

Algorithms	Test Set Accuracy
XGBoost	99.39%
SVM	55.86%
K-nearest Neighbor	70.78%
Logistic Regression	55.86%
Random Forest	84.38%
ANN	58.22%
CNN	81.76%

**Table 6:** Comparison of diagnostic accuracy rates among various algorithms.

Algorithms	Trend Component	Time-Domain Feature	Frequency-Domain Feature	Single-Scale Time Series Decomposition Algorithms	The Method Proposed in This Paper
XGBoost (TFTF)	76.44%	99.23%	89.35%	98.58%	99.39%
SVM	70.03%	55.85%	52.36%	55.68%	55.86%
K-nearest Neighbor	72.44%	67.88%	56.77%	67.67%	70.78%
Logistic Regression	49.94%	55.86%	59.05%	55.68%	55.86%
Random Forest	76.10%	80.12%	72.62%	83.40%	84.38%
ANN	49.99%	55.17%	51.66%	54.50%	58.22%
CNN	70.23%	77.66%	66.43%	78.63%	81.76%

**Table 7:** Correspondence between the number of removed features and the removed features.

Number of Removed Features	Removed Features
0	None
1	F17
2	F17, F4
3	F17, F4, F18
4	F17, F4, F18, F8
…	…
17	F17, F4, F18, F8, F6, F15, F3, F16, F5, F9, F2, F1, F11, F13, F12, F7, F14

## Data Availability

The original contributions presented in this study are included in the article. Further inquiries can be directed to the corresponding author.

## Appendix A

### Impact of Dataset Size on Model Robustness

The proposed model was trained and tested on subsets of the dataset with varying sizes (10%, 25%, 50%, and 75% of the original size). As shown in Table A1, the experimental results demonstrate that the method maintains high performance even on smaller datasets, with the accuracy decreasing by no more than 5%. These findings highlight the robustness of the proposed method and its applicability to scenarios with limited data availability.

**Table A1:** Effect of dataset size on accuracy.

Size of Dataset	Accuracy (%)
10% of the original size	95.56
25% of the original size	95.50
50% of the original size	96.99
75% of the original size	94.46

## Appendix B

### Robustness of the Model to Different Noise Levels

To further evaluate the model’s adaptability to noisy data, we designed experiments with input data under different noise levels, including Gaussian noise at 10%, 50%, and 80%. Here, 10% indicates that the noise range corresponds to 10% of the variability in the original data. The experimental results, presented in Table A2, show that as the noise level increases, the model’s performance fluctuates by less than 5%. This demonstrates the model’s strong robustness to noise.

**Table A2:** Effect of noise level on accuracy.

Noise Level	Accuracy (%)
0	99.39
10%	95.37
50%	95.17
80%	96.00

## Appendix C

### Effect of the Pooling Core Size on the Fault Detection Performance

The design involved six sets of experiments to evaluate the impact of different pooling kernel sizes on fault detection performance. These experiments covered a wide range of kernel sizes to assess the robustness of the model under various conditions:

Experiment 1: Tested very small kernels {1001, 2001, 5001}.

Experiment 2: The current baseline kernels {10001, 20001, 30001} were used.

Experiment 3: Expanded to intermediate ranges {15001, 25001, 35001} to explore the performance in moderate and slightly larger ranges.

Experiment 4: Evaluated larger kernels {50001, 70001, 90001}.

Experiment 5: Extremely small kernels {101, 301, 501} are tested to validate the model’s robustness under extreme conditions.

Experiment 6: Assess extremely large kernels {100001, 150001, 200001} for further robustness evaluation.

In Table A3, the baseline pooling kernels ({10001, 20001, 30001}) achieved the highest accuracy of 99.39%, whereas the intermediate-range kernels ({15001, 25001, 35001}) showed a decrease in accuracy to 95.79%, indicating the model’s sensitivity to overly moderate pooling ranges. In Experiment 1, when smaller kernels ({1001, 2001, 5001}) were used, the accuracy reached 98.53%, but excessively small kernels may result in insufficient information extraction. In Experiment 4, the larger kernels ({50001, 70001, 90001}) yielded an accuracy of 94.33%, suggesting that overly large kernels could lead to excessive smoothing. The extreme kernel sizes tested in Experiments 5 and 6 demonstrated some robustness, achieving accuracies of 97.06% and 98.11%, respectively, although neither outperformed the baseline.

**Table A3:** Accuracy for Different Pooling Kernel Sizes.

Experiment	Pooling Kernel Sizes	Accuracy (%)
Exp1	{1001, 2001, 5001}	98.53%
Exp2	{10,001, 20,001, 30,001}	99.39%
Exp3	{15,001, 25,001, 35,001}	95.79%
Exp4	{50,001, 70,001, 90,001}	94.33%
Exp5	{101, 301, 501}	97.06%
Exp6	{100,001, 150,001, 200,001}	98.11%
